# Phase Stability and Properties of Ti-Nb-Zr Thin Films and Their Dependence on Zr Addition

**DOI:** 10.3390/ma11081361

**Published:** 2018-08-06

**Authors:** Jeonghyeon Yang, Munkhbayar Baatarsukh, Joohyeon Bae, Sunchul Huh, Hyomin Jeong, Byeongkeun Choi, Taehyun Nam, Jungpil Noh

**Affiliations:** 1 Department of Mechanical System Engineering, Gyeongsang National University, 38, Cheondaegukchi-gil, Tongyeong 53064, Korea; jh.yang@gnu.ac.kr; 2 Department of Energy and Mechanical Engineering and Institute of Marine Industry, Gyeonsang National University, 38, Cheondaegukchi-gil, Tongyeong 53064, Korea; mundag9911@gmail.com (M.B.); jhyeonb@gnu.ac.kr (J.B.); schuh@gnu.ac.kr (S.H.); hmjeong@gnu.ac.kr (H.J.); bgchoi@gnu.ac.kr (B.C.); 3 Department of Materials Engineering and Convergence Technology & RIGECT, Gyeonsang National University, 501, Jinju-daero, Jinju 52828, Korea; tahynam@gnu.ac.kr

**Keywords:** Ti-Nb-Zr ternary alloys, porous structure, biomedical, Young’s modulus

## Abstract

Ternary Ti-Nb-Zr alloys were prepared by a magnetron sputtering method with porous structures observed in some of them. In bulk, in order to control the porous structure, a space holder (NH_4_HCO_3_) is used in the sintering method. However, in the present work, we show that the porous structure is also dependent on alloy composition. The results from Young’s modulus tests confirm that these alloys obey d-electrons alloy theory. However, the Young’s modulus of ternary thin films (≈80–95 GPa) is lower than that for binary alloys (≈108–123 GPa). The depth recovery ratio of ternary Ti-Nb-Zr thin films is also higher than that for binary β-Ti-(25.9–34.2)Nb thin film alloys.

## 1. Introduction

A low Young’s modulus and biocompatibility are two important criteria for biomedical applications [1]. Stainless steel and Co-Cr alloys have widely been used as metallic biomaterials for orthopedics and implants due to their favorable mechanical properties and thermal stability. However, the Young’s modulus of these alloys (≈210–240 GPa) is much higher than that of human bone (≈10–30 GPa). The high Young’s modulus results in bone stress shielding, which is harmful to human health [2]. Ti-Ni alloys have been extensively applied for biomedical uses to date, but it has been pointed out that pure Ni is a toxic element and can cause Ni-hypersensitivity [3]. Over the past decade, efforts have been made to remove cytotoxic nickel elements from biomaterials and replace them with non-toxic and allergy-free biocompatible elements [4]. β-type titanium alloys have attracted attention for biomedical applications because of their low stiffness, good corrosion resistance, biocompatibility, and superelasticity [5,6]. The β-type Ti-based alloys exhibit two stable phases, the β phase (bcc) and the α phase (hcp), along with exhibiting a number of metastable phases including α′ (hexagonal), α″ (orthorhombic), and ω phases [7]. The volume fraction of the β phase can be enhanced in titanium alloys by β-stabilizing transition metal (TM) elements such as Mo, V, Nb, Cr, Zr, and Ta [8].

Tantalum and niobium are considered the most potent β stabilizers and effectively reduce the modulus of elasticity of titanium alloys. On its own, zirconium is considered a neutral and weak β stabilizer. However, this element begins to act as a more potent β stabilizer by playing a role in binding to Nb [9]. These alloys are much costlier than pure titanium and Ti-6Al-V, owing to the addition of expensive Nb and Ta. In addition, large amounts of heavy elements, such as Nb, Mo, and Ta, lead to considerable increases in the density of β-type titanium alloys. Therefore, research and development of titanium alloys that are low cost, have a low Young’s modulus, and are light-weight are of high importance for biomedical applications [10,11]. The Ti-Nb-Zr alloy system has proved to be a good substitute for developing absolutely safe Ni-free biomedical Ti alloys. Ti, Nb, and Zr are non-toxic elements and thus do not cause any adverse reaction in the human body [12]. The addition of Zr to the β-type Ti-Nb alloy is beneficial for increasing the superelasticity, enhancing corrosion resistance, the maximum recovered strain, and the critical stress for slip deformation compared with Ta, and thus shows promising potential for biomedical applications [13,14,15,16].

An alternative to obtaining the desired smaller elastic modulus is to engineer porous structures, such as mimicking those in bones. Such porous structures reduce the elastic modulus due to their inherent geometry. In general, pores inside porous alloys consist of two types, either interconnected pores (open pores) or closed pores. Pores that are interconnected are more beneficial, as these enable nutrition delivery and body fluid transport in vivo [17,18]. In the present study, which is based on our previous experimental work examining binary Ti-Nb thin films, we investigate the addition of Zr and its influence on the phase stability and mechanical properties of Ti-Nb-Zr thin films.

## 2. Materials and Methods

### 2.1. Growth of Thin Film

Ti-Nb-Zr thin films were prepared on a glass substrate by magnetron sputtering, using pure metallic Ti, Nb, and Zr targets (2 inch size) at 773 K. Sputtering conditions were: Ar gas flow rate 20 SCCM, initial pressure 6.67 × 10^−4^ Pa, substrate temperature 773 K, and deposition time 120 min. Table 1 summarizes the growth conditions and compositions of the ternary Ti-Nb-Zr alloys. The distance between the target and the substrate was 80 mm.

In order to control the composition, the Ti target power was maintained at a fixed value of 200 W, while the DC powers for the Nb and Zr targets were varied from 60–85 W and 7–35 W, respectively. The working pressures were different for each deposited thin film. The reason for adjusting the working pressures is that the Zr target DC power is not stabilized during the addition of Zr, thereby causing problems in plasma formation. To compensate, the plasma is maintained by changing the working pressures. Experiments confirmed that changes in working pressure improve the deposition rate but do not affect the composition. Table 1 lists the growth conditions and the resulting compositions of the Ti-Nb-Zr alloys produced.

### 2.2. Characterization of Thin Films

The compositions of the Ti-Nb-Zr thin films were analyzed by energy dispersive spectroscopy (EDS, Jeol, JSM-6710F, Tokyo, Japan) at an accelerating voltage of 15 kV. The cross sections and surface features of the electrodes were characterized using field emission scanning electron microscopy (FE-SEM, Jeol, JSM-6710F) at 5 kV and 15 kV. The phase structures of the thin films were confirmed by X-ray diffractometry (XRD, Rigaku, Miniflex, Tokyo, Japan). XRD measurements were performed using Cu Kα radiation (λ = 1.5406 Å). Scans were performed over a 2θ range of 30–85° at a speed of 0.03°/s. Phase identification was made by comparing the obtained diffraction patterns with the JCPDS (Joint Committee on Powder Diffraction Standards) references. The mechanical properties of the Ti-Nb-Zr thin film specimens were investigated by nanoindentation tests (Fischerscope, HM2000, Windsor, CA, USA) at room temperature, using a Berkovich diamond indenter to a penetration depth of 325 nm.

## 3. Results

### 3.1. Surface and Cross-Section Characteristics of Thin Film

**Figure 1:**
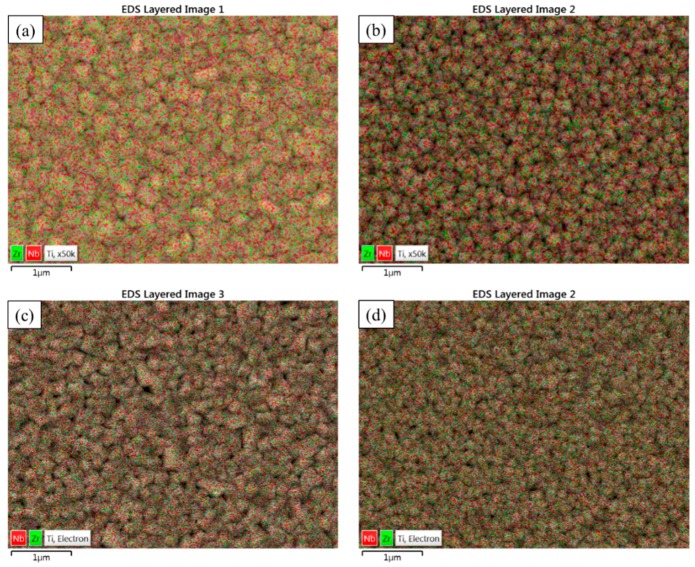
EDS layered image of ternary thin films of (**a**) Ti-22.8Nb-3.6Zr; (**b**) Ti-20.8Nb-5.6Zr; (**c**) Ti-18.3-8.9Zr; and (**d**) Ti-20.6Nb-12.7Zr.

It can be seen in Figure 1 that by using a stable plasma and constant substrate rotation speed (30 rpm), homogenous Ti-Nb-Zr thin films can successfully be deposited on the substrates. In these mapping images of Ti-Nb-Zr ternary alloys, grey spots correspond to elemental Ti, red spots to Nb, and green spots to Zr.

**Figure 2:**
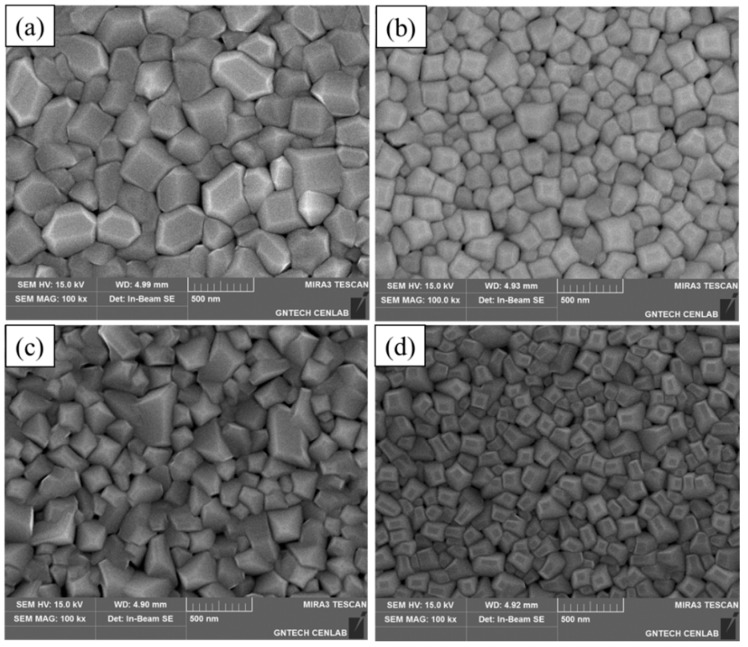
FE-SEM image of (**a**) Ti-22.8Nb-3.6Zr; (**b**) Ti-20.9Nb-5.6Zr; (**c**) Ti-18.3Nb-8.9Zr; and (**d**) Ti-20.6Nb-12.7Zr thin films.

SEM images of the Ti-Nb-Zr films exhibited different morphological properties. The dependence of grain size on alloy content is shown in the SEM images of Figure 2. For Ti-22.8Nb-3.6Zr (at.%), the grain size was 180 nm, and for Ti-20.9Nb-5.6Zr (at.%) it was 125 nm. The final two alloys with higher Zr contents have grain sizes of 150 nm (Ti-18.3Nb-8.9Zr (at.%)) and 94.2 nm (Ti-20.6Nb-12.7Zr (at.%)). Therefore, there was no simple relation between grain size and Zr contents.

**Figure 3:**
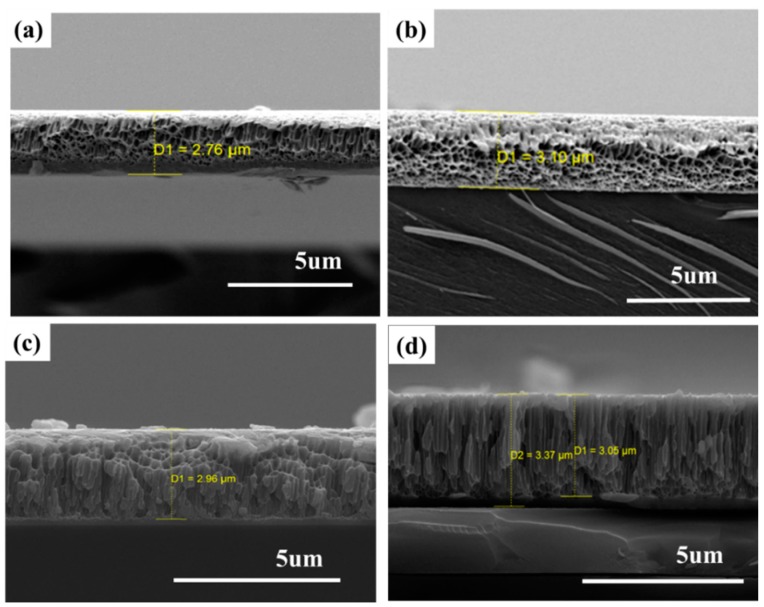
Cross-sectional SEM images of Ti-Nb-(3.6–12.7)Zr thin films: (**a**) Ti-22.8Nb-3.6Zr; (**b**) Ti-20.9Nb-5.6Zr; (**c**) Ti-18.3Nb-8.9Zr; and (**d**) Ti-20.6Nb-12.7Zr.

The cross-sectional images of the Ti-Nb-Zr films also exhibit different microstructures. In bulk, in order to control porous structure, a space holder (NH_4_HCO_3_) is used in the sintering method. The size of space holder particles was selected at around ≈20–800 µm [18]. The pores are thus strongly affected by the original space holder. In the present study, the samples had irregular and regular interconnected porosity structures, with micropores of several nanometers in size, observed in both Ti-22.8Nb-3.6Zr and Ti-20.9Nb-5.6Zr thin films. These are shown in Figure 3a,b. However, porous structures were not exhibited in Ti-18.3Nb-8.9Zr and Ti-20.6Nb-12.7Zr thin films, in-line with our expectation that the formation of porous structures is dependent on composition in such thin films.

The porosity of the deposited Ti-Nb-Zr thin films was calculated using Equation (1)

P = (1 − ρ/ρ_s_) × 100% (1)

where ρ is the density of the porous alloys, which can be determined by the weight and volume of the deposited samples (dimensions of 13.8 mm × 25.25 mm × 2.76 µm and 13.9 mm × 23.8 mm × 3.1 µm) and ρ_s_ corresponds to the theoretical (maximum) density. The theoretical density is 5.51 g/cm^3^ and 5.47 g/cm^3^ based on alloy compositions of Ti-22.8Nb-3.6Zr and Ti-20.8Nb-5.6Zr, respectively. The porosity of the deposited thin films were 2.81% and 7.30%, respectively. Note that these relatively low densities thus equate to high levels of porosity [19]. The porosity is expected to influence the mechanical properties of the resulting biomaterials, while simultaneously enhancing osseointegration [4].

### 3.2. Crystal Structure of Thin Film

**Figure 4:**
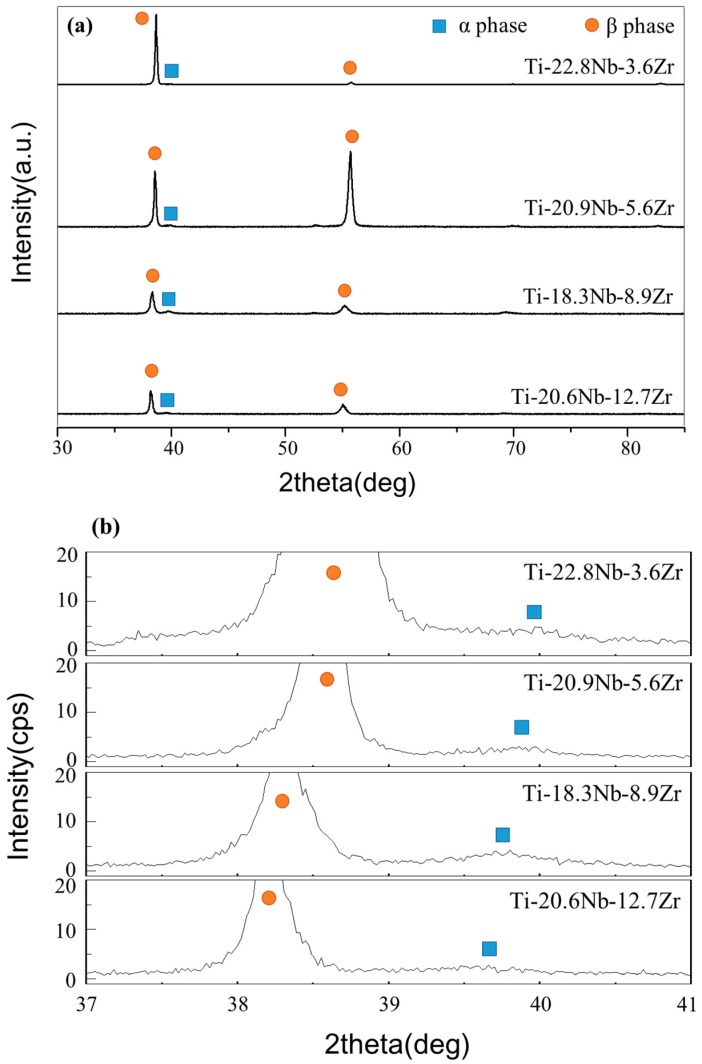
XRD patterns of Ti-Nb-(3.6–12.7)Zr (at.%) thin films (**a**); and a magnification of the range 37–41° (**b**).

Figure 4 shows XRD profiles for the ternary Ti-Nb-Zr alloys. Diffraction peaks correspond to the β and α phases in all alloys. In the microstructure of the all specimens, a small amount of α phase was identified within the predominant β phase. However, the temperature at which the martensitic (α phase) started to form (M_s_) decreased by 38 K with a 1 at.% increase of Zr content, and by 40 K with a 1 at.% increase of Nb content. Therefore, M_s_ of the Ti-22Nb-4Zr alloy was closest to room temperature [14]. This is comparable with the previous experimental results derived in binary Ti-Nb alloys, which indicate that all phases are β-type for Nb levels above Ti-25.5Nb (at.%). This suggests that the addition of Zr stabilizes both α and β phases in titanium alloys, but does not considerably influence the formation of α phases in Ti-Nb-Zr alloys. It was found that with increasing Zr addition, all diffraction peaks of β phase shifted to a low angle direction, suggesting expansion of the lattice from Zr [10]. The peak intensity of the β phase was higher than that of the α phase. However, addition of Zr resulted in a decrease in the intensity of the diffraction peaks, while peak width increased due to the accumulation of mechanical strain and reduction in crystallite size [1].

### 3.3. Superelastic Depth Recovery Ratio of Thin Film

**Figure 5:**
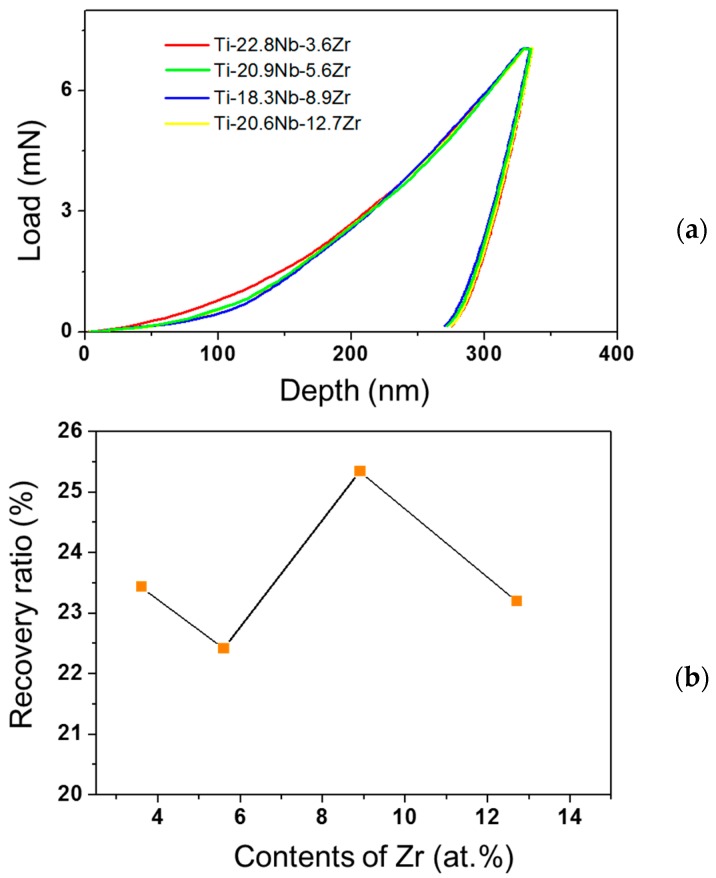
Nanoindentation L-D curves (**a**); and evolution of the depth recovery ratio (**b**) of Ti-Nb-Zr thin films.

Figure 5 shows nanoindentation load-displacement curves and depth recovery ratios of the Ti-Nb-Zr thin films, obtained by nanoindentation tests at room temperature. The loading was carried out to a depth of 325 nm before removal. The superelastic recovery depth ratio was calculated from the load-displacement curves using

η = W_rc_/W_t_ (2)

where W_t_ is the total deformation energy during the indentation process, and W_rc_ is the recoverable deformation energy. The highest superelastic recovery ratio was exhibited in the Ti-18.3Nb-8.9Zr thin film. The depth recovery ratio was 22.4–25.3% for the ternary Ti-Nb-(3.6–12.7)Zr alloys, higher than that observed in our previous work of 15.5–17.5% for β-type Ti-(25.9–34.2)Nb alloys [20]. This means that the addition of Zr was effective to increase recovery strain.

### 3.4. Young’s Modulus and Hardness of Thin Film

**Figure 6:**
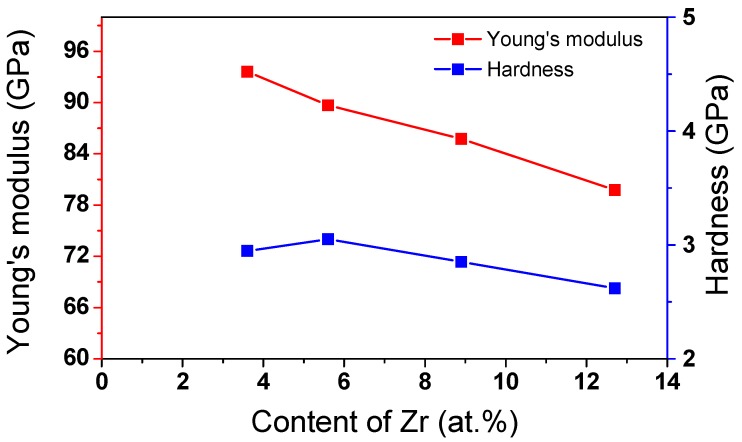
Dependence of the Young’s modulus and hardness on the Zr content.

It can be seen in Figure 6 that the Young’s modulus decreases with increasing Zr content between Ti-22.8Nb-3.6Zr and Ti-20.6Nb-12.7Zr. The *d*-electrons alloy theory is an effective method of designing titanium alloys with a controlled Young’s modulus [12]. Young’s modulus is an intrinsic feature of materials, determined by the interatomic bonding force. Transition metals have special *d*-electrons, which tend to form covalent bonds. Essentially, the bonding force of metallic bonds is derived from Coulomb electrostatic interactions among valence electrons and positive ion cores. The interatomic bonding force of titanium alloys can be estimated by combining Coulomb’s law and the *d*-electrons alloy theory, as in Equation (3):

(3) $$\mathrm{bonding}\;\mathrm{force}=\frac{Z_{\mathrm{eff}}+B_{o}}{M_{d}^{2}}$$

where Z_eff_ is the effective nuclei charges, B_o_ is a measure of the shared electron density, and M_d_ is the energy level of the d orbit of an alloying element M (more details are given in Refs. [21,22,23]).

B_o_ is a measure of the covalent bond strength between Ti and an alloying element M, and M_d_ correlates with electronegativity and the metal radius of elements. The Young’s modulus decreased with the addition of Zr elements for the ternary alloys, and B_o_ and M_d_ increased between Ti-22.8Nb-3.6Zr and Ti-20.6Nb-12.7Zr, as shown in Table 2. According to this theory, low bonding forces are related to a low Young’s modulus. In this case, the bonding force of 1.68 corresponded to a minimum Young’s modulus of 79.78 GPa for Ti-20.6Nb-12.7Zr alloy. The results for the Young’s modulus confirmed that the *d*-electrons alloy theory holds for these ternary alloys.

According to the Hall-Petch relationship, the yield strength is dependent on the grain size, which in turn corresponds to hardness for Vickers indentation. Therefore, any increase in grain size of the samples could result in a decrease in hardness [24]. However, in the current study, the hardness decreased with decreasing average grain size. The hardness and average grain size of Ti-22.8Nb-3.6Zr, Ti-20.9Nb-5.6Zr, Ti-18.3Nb-8.9Zr, and Ti-20.6Nb-12.7Zr alloy were 2.95 GPa, 3.05 GPa, 2.85 GPa, and 2.62 GPa and 180 nm, 125 nm, 150 nm, and 94.2 nm, respectively. Therefore, the change of hardness could not be simply explained by the Hall-Petch relationship. Further studies will be needed to determine the relationship between the hardness and change of Zr contents.

## 4. Discussion

The effect of adding Zr on the phase stability and mechanical properties of Ti-Nb and Ti-Nb-Zr films was investigated. Table 3 compares the properties of the binary Ti-22.6Nb thin film alloy [20] with those of the ternary Ti-22.8Nb-3.6Zr thin film alloy of the current study. The additional Zr content did not change the crystallographic structure in binary and ternary alloys, and both the α and β phases appeared in both. However, mechanical properties, such as Young’s modulus and hardness, decreased from 116 GPa to 95 GPa and from 5.3 GPa to 2.9 GPa, respectively, and the depth recovery ratio increased from 19.6% to 23.5% with the addition of Zr content.

Table 4 compares the properties of the ternary Ti-20.9Nb-5.6Zr thin film alloy with those of the ternary Ti-20.6Nb-12.7Zr one. The additional Zr content did not change the crystallographic structure, and again α and β phases appeared in both alloys. The Young’s modulus decreased from 89.7 GPa to 79.8 GPa, and the depth recovery ratio increased from 22.4% to 23.2% with further Zr added, while in contrast, the hardness decreased as the Zr content rose.

## 5. Conclusions

Ternary Ti-Nb-(3.6–12.7)Zr (at.%) thin films were prepared using magnetron sputtering. The effect of adding Zr content on the phase stability and mechanical properties of the Ti-Nb-Zr films was investigated. The addition of Zr does not change the crystallography in the ternary alloy, and α and β phases appeared in the alloys. This suggests that the addition of Zr stabilized both α and β phases in titanium alloys, and did not considerably influence the formation of α phases in Ti-Nb-Zr alloys. The Young’s modulus decreased from 94.65 GPa to 79.78 GPa in ternary alloys with additional Zr content. The results of the Young’s modulus tests are in agreement with *d*-electrons alloy theory for these ternary alloys. However, the Young’s modulus of ternary thin films (80–95 GPa) was lower than that for binary alloys (108–123 GPa). We opine that the high hardness established in Ti-20.8Nb-5.6Zr was related to the porous structure of such ternary alloys. The high depth recovery ratio of 22.4–25.3% for the Ti-Nb-(3.6–12.7)Zr alloys indicates that addition of Zr was effective in increasing recovery strain.

## Figures and Tables

**Table 1:** Growth condition and composition for Ti-Nb-Zr thin film.

No.	Ternary	Ti Power (W)	Nb Power (W)	Zr Power (W)	Working Pressure (Pa)	Composition (at.%)
1	Ti-Nb-Zr	200	80	7	0.867	Ti-Nb_22.8_-Zr_3.6_
2		200	70	15	0.560	Ti-Nb_20.9_-Zr_5.6_
3		200	60	25	0.560	Ti-Nb_18.3_-Zr_8.9_
4		200	65	35	0.267	Ti-Nb_20.6_-Zr_12.7_

**Table 2:** Relationship between bonding force and Young’s modulus.

No.	Ternary	Z_eff_	Bonding Force	Young’s Modulus, GPa
1	Ti-22.8Nb-3.6Zr	3.49	1.76	94.65
2	Ti-20.9Nb-5.6Zr	3.35	1.75	89.69
3	Ti-18.3Nb-8.9Zr	3.30	1.73	85.75
4	Ti-20.6Nb-12.7Zr	3.25	1.68	79.78

**Table 3:** Mechanical properties of ternary alloy compared with binary alloy.

Binary	Phase Constitution of α, β Phase	Ternary
Ti-22.6Nb		Ti-22.8Nb-3.6Zr
19.60%	Superelastic depth recovery ratio	23.5%
116 GPa	Young’s modulus	95 GPa
5.3 GPa	Hardness	2.9 GPa

**Table 4:** Effect of Zr content on crystallographic and mechanical properties of ternary alloys.

Ternary	Phase Constitution of α, β Phase	Ternary
Ti-20.9Nb-5.6Zr		Ti-20.6Nb-12.7Zr
22.40%	Superelastic depth recovery ratio	23.20%
89.7 GPa	Young’s modulus	79.8 GPa
3.05 GPa	Hardness	2.62 GPa
